# Effects of Solar Radiation on the Eyes

**DOI:** 10.7759/cureus.30857

**Published:** 2022-10-29

**Authors:** Dishita Chawda, Pranaykumar Shinde

**Affiliations:** 1 Department of Ophthalmology, Jawaharlal Nehru Medical College, Datta Meghe Institute of Medical Sciences, Wardha, IND

**Keywords:** ultraviolet radiation, eye cancers, outdoor work, solar rays unearthing, macular degeneration, cataract

## Abstract

It is known that exposure to solar radiation (SR) can cause various harmful effects on one's health, most of which are related to the ultraviolet (UV) component of SR. A considerably high number of people around the world who work outside are constantly exposed to SR for the majority of their working lives. The eye is the primary organ affected by short-term and long-term exposure to solar radiation. According to an ever-growing body of research, cataracts, pterygium, and macular degeneration are all possible side effects of prolonged exposure. Despite this, the danger of SR exposure is presently underestimated, if not completely overlooked, as a component of occupational risk for employees who do their duties outside. SR exposure is impacted by a wide range of individual and environmental factors; nevertheless, occupational exposure is among the most significant. The scarcity of affordable and accepted methods to measure SR worker exposure, particularly long-term exposure, is one of the key obstacles that must be overcome before a more excellent knowledge of this risk and the development of more effective preventive strategies can be accomplished. This review was conducted with the primary objectives of providing a comprehensive overview of the SR exposure risk of outdoor workers, including UV exposure extents and the chief approaches recently proposed for short-term and cumulative exposure, as well as providing an update on the information presented regarding the most common adverse eye effects. In conclusion, this article will be presenting preventative steps that may be taken to reduce occupational risk.

## Introduction and background

There is a possibility that exposure to solar radiation (SR) at work is the earliest known occupational danger. The earliest instances of contamination happened when early people participated in activities such as hunting and fishing and then again when agriculture became an everyday activity [1]. Solar radiation is a hazard that can be viewed as a potential occupational risk; the sun emits a wide variety of energy wavelengths across the entire electromagnetic spectrum, but primarily in the range of non-ionizing radiations, which includes the vast majority of naturally occurring incoherent optical radiation. Solar radiation is a potential threat to people exposed to it in their line of work. In most instances, radiation can only penetrate biological tissues to a very shallow depth. However, each wavelength is distinct in terms of its capacity to interact with live beings and penetrate biological tissue, the processes engaged, and the potential ramifications that may result. When compared to the spectrum of solar radiation emitted by the sun, the solar radiation spectrum received at the Earth's surface is quite different. This is because various components of the atmosphere have a shielding effect. The ozone layer can absorb light with wavelengths shorter than 290 nanometers at their longest point [2]. However, due to the depletion of the ozone layer, a higher amount of ultraviolet B (UV-B) radiation is making its way to the surface of the globe. This may have adverse health effects. Because of these factors, during this fundamental research, a focus was placed on the dangers linked with exposure to UV radiation (UVR) from the Sun [3]. 

The primary purpose of this review is to analyze the occupational solar radiation exposure risk posed to the workers who are required to carry out their responsibilities outdoors.

## Review

Mechanism and pathophysiology

Solar radiation has the potential to interact with biological tissues through two primary mechanisms: photochemical, which is typical of wavelengths of UV light, and thermal, which is the predominant process when it comes to wavelengths of infrared light. Both of these mechanisms are discussed further below.

The photochemical effect predominates in the wavelengths corresponding to violet-blue light. In contrast, the thermal effect dominates in the spectrum region corresponding to yellow-red light [3]. Both processes may be recognized in the visible section of the SR spectrum. Neomelanogenesis and skin thickening are the two primary responses of the skin to UV light exposure. Sun tanning is the common name for the darkening of the skin that occurs as a consequence of these reactions, which are regarded to constitute an adaptive defense mechanism [4]. Long-term exposure leads to the complicated phenomena known as photo-aging, which is associated with multiple distinct UV components but is caused mainly by the persistent damage from UV-A.

The human eye has several distinct ocular structures, each responsible for the absorption of a particular SR band. This absorption, in turn, causes a range of thermogenic and photochemical effects. On the other hand, the cornea and the lens are both responsible for absorbing UV-B and UV-A rays. It is important to note that around 1-2 percent of near UV-A may reach the retina; age-related alterations with quantities as high as 10 percent in younger people have been documented. The retina is responsible for the absorption of the visible spectrum and near-infrared light [5].

Factors Influencing solar UV radiation exposure to the eyes

**Figure 1:**
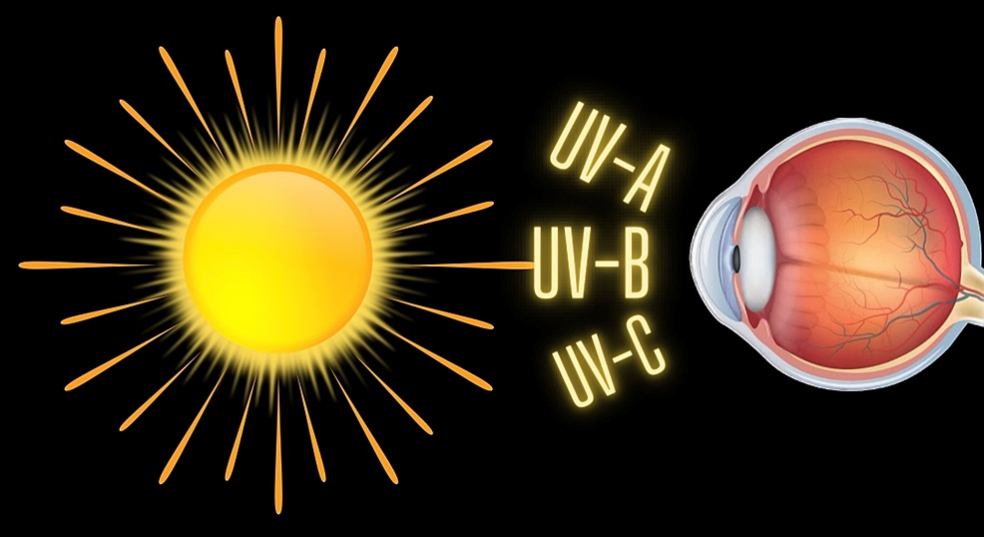
Different UV rays on the eyes UV = ultraviolet Image credit: Author Dishita Chawda

The following is a list of the elements which influence solar UV radiation exposure to the eyes, as provided by International Commission on Non-Ionizing Radiation Protection (ICNIRP): 1. Pollutants: The presence of pollutants in the air cuts down on UV exposure. 2. Latitude: The amount of undiscovered UV rays is proportionally reduced with increasing latitude away from the equator. 3. Altitude: At higher elevations, more of the Sun's UV rays are exposed to the atmosphere. 4. Reflection: The reflection of surfaces in the environment may play an essential role in the amount of solar UV radiation an individual is exposed to. This phenomenon can potentially increase UV radiation exposure in parts of the body that are usually protected from direct sunlight, such as the eyes. A unique aspect of albedo is referred to as the Coroneo effect, and it occurs when light rays coming from the temporal side of the face (Figure 1) are refracted by the corneal dome in the nasal corneal limbus as well as in the nasal and inferonasal regions of the lens [6]. 5. Personal considerations: The execution of the outside activity, whether at work or during leisure time on vacations or for the practice of sports or outdoor pastimes, is one of the personal factors that might influence a person's exposure to the Sun's UV radiation. Individual behaviors, such as covering up with protective clothes, sunglasses, and hats, protecting oneself with sunscreen, and seeking out shade, are among the most critical factors determining the amount of solar UV exposure [7]. 6. Occupational considerations: Work performed outside is particularly significant in cumulative exposure; photochemical damage will likely accumulate in workers' eyes for their employment, ultimately leading to detrimental consequences.

Adverse health effects of solar rays 

Pterygium

It is a wing-shaped growth of the conjunctiva onto the cornea that can cause ocular discomfort, aesthetic consequences, and, in the late stages of corneal tissue invasion, vision impairment. Pathogenesis is still not known. A fourfold risk has been seen in regions where solar rays are elevated. Occupational exposure was positively linked with illness severity. Sunglasses and hats are found to be influential factors in preventing pterygium.

Cataracts

In every region of the world, cataracts are the leading cause of blindness. Exposure to UV light over an extended period is one of the most critical risk factors [8]. The incidence of cataracts has decreased as a direct result of the lessening of the Sun's direct beams [7]. The most recent evidence for a causal relationship between occupational SR exposure and cataracts came from research on the nuclear subtype of the condition [8].

Macular Degeneration (MD)

It is a disorder that damages the macula and causes progressive vision loss. This vision loss often starts in the center of the visual field and progresses outward [3]. The advancement is slow, taking many years to produce considerable visual impairment, and the severity of the condition varies from the early to the late stages of the illness. The disease progresses in phases. It is also the primary cause of vision impairment in nations such as the United Kingdom and the United States [8]. A shift in the metabolic sustainment of photoreceptor cells (rods and cones) and the retinal pigment epithelium (RPE) as a consequence of inflammatory processes and vascular abnormalities is suggested to be the source of long-lasting retinal damage in MD. According to the World Health Organization, prolonged exposure to SR, in particular its blue light component, is a risk factor for MD. Other risk factors include smoking, diabetes, genetics, and alcohol abuse [9].

Photokeratitis

Photokeratitis is induced by sudden exposure to UVR, and the symptoms typically go away between 8 and 12 hours after the exposure. Symptoms such as significant vision loss, sensitivity to light, and severe eye pain are brought on by irritation and destruction to the cells that make up the superficial layer of the epithelium that lines the cornea [10]. Photosensitivity may be caused by prolonged exposure to bright light, whether it comes from the Sun or a fluorescent light bulb. Snow blindness is induced by natural UVB exposure and happens when the light is substantially reflected, such as while skiing or in high mountains. Even momentary exposure to UVB and UVC rays may result in a painful condition known as the welder's eye, which is a form of photokeratitis [11]. 

Climatic Droplet Keratopathy (CDK)

The development of concretions in the superficial layer of the cornea, often referred to as spheroidal degeneration, is one of the defining characteristics of CDK. Because countries in the arctic and tropical regions are subjected to the most significant amounts of UVR, the condition is more prevalent in those areas. It is commonplace in the parts that are subjected to the highest levels of UVR, such as the arctic and tropical regions [9]. It is generally agreed upon that CDK may be attributed to prolonged exposure to UVA and UVB rays. 

Squamous Cell Carcinoma (SCC) of the Conjunctiva

UVB radiation is believed to be the primary cause of SCC of the conjunctiva and cornea; viruses human papillomavirus (HPV) and HIV are also likely to be related to the disorder. SCC of the conjunctiva was seen with a high frequency in the Ugandan population that is close to the equator. Studies on populations suggest that there is a connection between the geographic distribution of conjunctival and corneal SCC incidence and the amounts of ambient solar radiation in the environment [12].

Photo Retinitis

Looking at the Sun during an eclipse, either directly or indirectly, is the most common cause of eclipse blindness, which is also referred to as acute photoretinitis. This is a very uncommon photochemical damage to the retina that is most often caused by staring at the Sun. It manifests itself with symptoms such as a decrease in visual acuity, scotomas in the visual field, and difficulty seeing items in one's environment. Vision acuity often recovers to normal levels between three to nine months [13-20].

However, there is always the possibility that an irreversible visual impairment may occur in certain circumstances. In the vast majority of instances, patients have a recovery to normal visual acuity. The National Aeronautics and Space Administration (NASA) says that to see an eclipse safely, all that is necessary is a pair of glasses designed expressly for the occasion [8]. Melanoma is detected in the eyeball more often than in any other region apart from the skin. Melanoma of the eyeball will nearly always impact the choroid, and when it does, it will almost permanently damage the ciliary body as well (85 percent of the time) [9,11]. The lenses of children's eyes are more transparent than the lenses of adult eyes and allow for a more significant amount of radiation to be transmitted to the retina. As a direct consequence, children are at a greater risk of developing cancer due to the carcinogenic effects of UV radiation [14].

Protection against solar radiation

Ski goggles, hats with wide brims, contact lenses with UV filters, glasses with coatings that block UV rays, sunglasses with UV filters, and skiing goggles are just some of the many options available for shielding one's eyes from the potentially damaging effects of the Sun's rays [15]. Other options include wearing glasses with coatings that block UV rays. It is to the user's advantage to link as many of these various channels all at once as possible. Nevertheless, the tactic that has shown to be the most effective is to avoid being in the Sun at those periods of the day when the Sun's intensity is at its highest [4]. Eye protection needs to be significant in mountainous locations with high elevations and in situations with powerful light reflections, such as when there is snow or sand present. This includes any environment where there is a combination of these two factors [16]. Contact lenses that are classified as class I are required to absorb at least 90 percent of UVA and 99 percent of UVB, while contact lenses that are classified as class II must absorb 70 percent of UVA and 95 percent of UVB [17,20-25]. These requirements were established by the American National Standards Institute. Contact lenses provide an extra layer of protection since they cover not only the cornea but also its limbs. This complete coverage of the cornea helps prevent infections. When a person wears dark sunglasses, their pupils do not contract, which stops the eyes' natural defense system from working [18,26-33]. As a result of this, it is of the utmost importance to always wear sunglasses that include a UV filter inside them. This will guarantee that an excessive amount of harmful wavelengths does not reach the eyes through the dilated pupils to protect them from damage [19,34]. The vast majority of sunglasses let sunlight that has been reflected into the eye from three distinct directions: from the top, from the bottom, and from the sides. It is essential to pay close attention to the shape of the sunglasses that you choose. Because they provide the highest level of all-around protection for the eyes, the ones that have a body that extends toward the temples are the ones that should be used. Experiments and studies have shown that wearing a hat with a wide brim may reduce the quantity of UV light that reaches a person's eyes by a factor of four [20,6].

## Conclusions

There are a variety of eye conditions that have been connected to UVR exposure. Short-term exposure to UVR has been shown to cause photokeratitis and photo retinitis. In contrast, long-term exposure to UVR has been shown to cause CDK, cataracts, pterygium, SCC of the cornea and conjunctiva, and cancers of the eyelids. The evidence supporting these claims is quite compelling. Conditions such as pinguecula, age-related macular degeneration, and melanoma of the eyeball are examples of eye diseases that may or may not have a link to UVR radiation. Because the destructive effects of UV radiation are cumulative, it is important to protect the eyes of young persons, who are more sensitive to the effects of ultraviolet light. This is because young individuals are more likely to be exposed to ultraviolet light. Despite appearances to the contrary, the amount of UV radiation that reaches the eyes does not decrease when there is cloudy weather present. The eyes are exposed to ultraviolet radiation around the clock every day of the year. Protecting oneself from the Sun may be accomplished in various ways, including using proper clothing, hats, glasses, or contact lenses that filter UV radiation.
